# “Nothing Comes to Mind…”: Challenges With Identifying One’s Own Role in Preventable Adverse Outcomes in Interprofessional Birthing Unit Teams, and the Implications for Quality Improvement Initiatives

**DOI:** 10.5334/pme.1651

**Published:** 2025-05-07

**Authors:** Lauren Columbus, Ayma Aqib, Rachael Pack, Harrison Banner, Taryn Taylor

**Affiliations:** 1 Department of Midwifery, London Health Sciences Centre, London, Canada; 2 Department of Family Medicine, Schulich School of Medicine & Dentistry, Western University, London, Canada; 3 Schulich School of Medicine & Dentistry, Western University, London, Canada; 4 Centre for Education Research & Innovation, Schulich School of Medicine & Dentistry, Western University, London, Canada; 5 Department of Obstetrics & Gynaecology, Schulich School of Medicine & Dentistry, Western University, London, Canada; 6 Department of Obstetrics & Gynaecology, London, Canada; 7 Centre for Education Research & Innovation, Schulich School of Medicine & Dentistry, Western University, London, Canada

## Abstract

**Introduction:**

Preventable adverse perinatal outcomes have a devastating impact on patients and providers and form the basis of many quality improvement (QI) and patient safety initiatives in birthing unit teams, including fetal health surveillance (FHS) training programs. Birthing unit staff attitudes regarding the role of interprofessional relationships on FHS decisions remain largely unexplored with respect to preventable adverse outcomes.

**Methods:**

In this intervention-primed, constructivist grounded theory study, members across all five professions providing intrapartum care at one academic centre attended an interprofessional workshop on improving their FHS interpretation, response, communication, and teamwork skills. Twenty-three birthing unit team members across midwifery, obstetrics, family medicine, nursing, and obstetrical trainees were purposively sampled and completed semi-structured interviews. Self-serving bias theory was used as a sensitizing concept to explore the social phenomena observed.

**Results:**

Birthing unit staff constructed a self-schema of their role in FHS management that was more flattering than the person-schema created by their colleagues about them. The schemas encoded four categories of information that included (1) Identifying the offender, (2) Assigning blame (3) Aligning with the “right” philosophy of care, and (4) Defending one’s profession. Participants demonstrated distorted perceptual processes where they described errors other team members had made with ease but struggled to acknowledge their own role in poor outcomes.

**Discussion:**

Dissonant schemas can be barriers to the accurate self-assessment of one’s skills and have significant implications for interprofessional team competence. QI initiatives may be of limited efficacy given these findings, but addressing these distorted perceptual processes in QI initiatives could improve team performance.

## Introduction

Adverse perinatal outcomes resulting from intrapartum hypoxic fetal events are a devastating occurrence for families and can significantly impact obstetrical care providers both emotionally as well as medico-legally. They also represent a significant portion of the litigation costs associated with the Obstetrics/Gynecology specialty in Canada [1]. To mitigate preventable adverse outcomes, intrapartum fetal health surveillance (FHS) methods are employed in most intrapartum settings. The most frequently suggested continuing professional development (CPD) intervention for FHS quality improvement (QI) is to provide more knowledge and technical skills training to birthing unit team members [2], but evidence for optimal content and method of delivery of this type of training program remains limited [3,4]. Health professions education (HPE) research focused on team training and communication interventions has also failed to demonstrate an impact on adverse perinatal outcomes [5,6,7], despite team communication being identified as a primary issue in cases of perinatal morbidity and mortality associated with abnormal intrapartum FHS [8]. There is a need for exploratory work about how the psychosocial influences on interprofessional birthing unit teams impact not only an individual’s decision-making and behaviour with respect to FHS, but also the team’s collective competence. This may help us design FHS training programs that can reduce adverse perinatal outcomes.

The collective competence of a team is fluid and ever evolving, because of the complexity of the social relations on a team [9]. Research on continuing professional development initiatives has shown that this is particularly true on the birthing unit, where teams can consist of upwards of five different professions (midwifery, obstetrics, family medicine, nursing, and obstetrical trainees), and where overlap in scope of practice can be significant [10,11]. The hierarchical model of interprofessional team decision making on birthing units can limit the use of unique skill sets across disciplines [12]. Divergent philosophies of care also influence the disparate ‘cognitive maps’ held by individuals in different health professions, as they reflect how each discipline is educated and socialized [13,14], and influence what each profession “sees”: individuals in different professions can look at the same information and yet not see the same thing [15]. Research in health professions education and quality improvement shows that this may contribute to the allegiances that individuals have towards their own profession rather than to the larger birthing unit team [14,16,17,18], which may create incompatible team goals [19] and inhibit information-sharing critical to the patient’s outcome [18].

Current conceptualizations of interprofessional education (IPE) have examined the ways in which previous iterations have fallen short of reaching their goals of creating better collaborators [20], and the lack of evidence linking IPE to improved patient outcomes [21]. IPE should include an explicit focus on teaching team skills, should take place in workplace-based settings, and should address professional hierarchies, power, and conflicting philosophies of care [21,22]. Traditionally, most FHS training programs are not delivered in interprofessional settings, and they continue to focus on knowledge and technical skills without fully addressing the myriad psychosocial influences that manifest in interprofessional teams [2,13,23,24]. Recent research suggests high-fidelity FHS case scenarios could be a promising intervention [10,25], but this fidelity can only be achieved when the training programs reflect the interprofessional reality of birthing unit teams, and the psychosocial complexity involved.

Our large tertiary care centre in Ontario, Canada, implemented interprofessional FHS refresher courses for all members of the obstetrical care unit staff in 2022, following recommendations from the Society of Obstetricians and Gynecologists of Canada’s clinical practice guideline on intrapartum FHS [26]. In Canada, FHS Refresher courses are national standardized courses designed for biennial attendance of all front-line intrapartum care providers. They include a combination of technical updates, interactive authentic case scenarios in which to practice FHS interpretation, and a didactic session on teamwork and communication, which take place across an online self-learning module as well as a four-hour in-person class session. The course content is updated in line with national clinical practice guidelines and includes recommendations from the Canadian Medical Protective Association and the Health Insurance Reciprocal of Canada based on adverse perinatal events. Course objectives include explaining maternal-fetal physiology and pathophysiology, discussion of clinical management of FHS interpretation, as well as “identifying strategies to mitigate risks through improved interdisciplinary communication, collaboration and management” [27]. While the courses were not implemented as part of this study, they provided an opportunistic moment to design an intervention-primed qualitative inquiry with the aim of building a conceptual understanding of the psychosocial approach to this training from an interprofessional group of front-line intrapartum care providers, since studies of this nature did not exist in the literature [3].

### Conceptual Framework

This study uses a social psychology theory—the self-serving bias theory—within our conceptual framework to examine the phenomena at play in FHS decision-making in the birthing unit team setting. Self-serving bias theory describes socially observed phenomena with well-established evidence in the social psychology field and offers a robust theory behind the cognitive and motivational processes humans employ in their response to errors and successes [28,29,30,31]. The theory holds that people willingly accept credit for their successes but will deny responsibility for failures for which they are equally liable [29,32]. Individuals will also make ultimate attribution errors towards outgroup members, which “may reflect the operation of the self-serving bias refracted through social identification” [33, p. 436]. Self-serving biases also impact how individuals receive and respond to feedback, and how people evaluate their performance relative to colleagues [29], which is particularly relevant to FHS training and preventable adverse outcomes but also to the success of quality improvement initiatives. Any bias, including self-serving biases, can impact the way we process information through our cognitive schemas [34]. Schemas have been defined in cognitive psychology as “knowledge structures that represent objects or events and provide default assumptions about their characteristics, relationships, and entailments under conditions of incomplete information” [35, p. 269]. Schemas simplify cognition and assist our brains in processing the significant amount of information we encounter [34]. But they can also cause erroneous thinking if new information does not align with an existing schema, and they may also cause people to inaccurately recall events [35].

The aim of the study is to explore how psychosocial influences on interprofessional teams impact FHS decision-making. In answering this question, we hope to understand how these findings can be applied to improve FHS training programs and reduce adverse perinatal outcomes.

## Methods

### Methodology & Reflexivity

We employed a constructivist grounded theory (CGT) methodology. As the research question focuses on the social, psychological, and cultural features that impact our approach to FHS decision-making, CGT best allows for the collection of a diverse range of perspectives across multiple professions and to actively collect data with our participants as we attempt to understand the phenomena at play. This CGT study was intervention primed, using the interprofessional FHS Refresher Course as a priming intervention which we describe below in Table 1. Priming a qualitative study builds on constructivist approaches in simulation research to enrich qualitative data by leveraging the emotional activation of an intervention (in our case, the simulated FHS case scenarios in the refresher courses) to prime participants to engage in more thoughtful dialogue regarding sensitive situations, such as those that involve discussions of power, clinical disagreements, speaking up, and hierarchies [36,37]. From the conception of the study, we have applied Lincoln & Guba’s trustworthiness criteria to ensure methodological rigour [38].

**Table 1:** Description of Priming Intervention and Methodology.


EDUCATIONAL INTERVENTION	PARTICIPANTS	SETTING	FHS CASE SCENARIOS AS PRIMING INTERVENTION

Twelve four-hour, mandatory interprofessional FHS Refresher courses, designed by the Canadian Perinatal Program Coalition, run from May through October 2022 All 260 birthing unit staff and employees at an academic, tertiary care centre in Ontario, Canada The courses had not previously been conducted on the birthing unit and had strict interprofessional composition (members present from all five disciplines on the birthing unit and instructors representing at least two different disciplines for each session).	The interprofessional team composition at this academic institution consists of five disciplines, which is typical of most academic hospitals in Canada: Nursing Obstetrics Family medicine-Obstetrics Midwifery Resident trainees in Obstetrics & gynaecology	A tertiary care centre was chosen because their birthing teams typically involve the widest range of professions and involve learners. LC and TT are certified FHS instructors and were involved in the delivery of the refresher courses at this institution.	Participants were primed with three simulated FHS case scenarios based on actual cases that involve challenging or ambiguous decisions regarding FHS decision making Insights generated from the FHS case scenarios in the courses and documented in field notes were used to inform the development of our interview guide, in addition to existing relevant literature. The field notes and insights from the courses were not used as part of the data analysis, but rather to assist with interview guide creation and sampling decisions.


Reflexivity constitutes an important aspect of confirmability [38]. LC acknowledges that as a registered midwife trained in a profession rooted in feminist healthcare ideology as it originated in Ontario, discussions of power and hierarchies in the healthcare system forms a central lens in her outlook regarding pregnancy care. Openly acknowledging this position in team meetings and in debriefing interviews and data analysis meetings was critical in the confirmability process, and a multidisciplinary research team allows for a richness and variety of perspectives. Two senior researchers, TT and HB are obstetrician-gynecologists and acknowledge their experience working previously as residents in a position of lesser power and currently as consultant obstetricians in a position of power within the obstetrical care team. RP, a feminist sociologist with an interest in health care relations, brought her perspective on power and hierarchy to bear on the research. Her perspective was informed by her outsider status as a non-healthcare provider. AA is a medical student in the early years of her training. LC, HB, and TT all had insider-status as members working within the same setting as the participants, with TT and HB holding evaluative relationships with residents. Thus, interviews were conducted by LC, RP, and AA and transcripts were de-identified and anonymized by removing identifying details of participants prior to being shared with HB and TT.

### Setting, Participants, and Design

Purposive sampling for the semi-structured interviews was conducted across the 260 members of the birthing unit, with the objective of maximizing variation in participant voices and including a diversity of perspectives. Using our observations and field notes from the refresher sessions, HB, LC, and AA requested interviews from participants who were identified as being willing to engage in reflection and candour during the refresher course, who provided interesting or diverse perspectives, or who represented individuals situated within differing tiers of the hierarchy of the birthing unit. During a one-on-one semi-structured follow-up interview, participants were invited to reflect on the social and system dynamics that affect FHS management (as raised in the FHS case scenarios during the course) and to consider how these aspects shape their real-life practice [39].

### Data Collection & Analysis

Participants (n = 23) across all five professional groups providing intrapartum care were sampled: obstetricians (OB, n = 3), obstetrics & gynaecology residents (OB-R, n = 4), registered midwives (RM, n = 7), family physicians who provide obstetrical care (FM-OB, n = 4), and registered nurses (RN, n = 5). Data collection occurred between June 2022 and June 2023. Semi-structured interviews of 45–60 minutes in duration were conducted based on interview guides. Our research team has elected not to provide information on the gender, race, age or experience levels of participants to reduce the possibility of identifying quotations, given the sensitive nature of the interview content. LC, AA, and RP conducted the interviews virtually using Zoom, which were audio recorded and transcribed verbatim. Both the FHS courses and interviews were held virtually due to the ongoing COVID-19 pandemic and our organizational restrictions in place at the time.

The de-identified transcripts were initially coded inductively, using line-by-line coding with gerunds by LC and AA in an iterative fashion using constant comparative analysis [40]. Reflexive self-analysis discussed in regular team meetings throughout the data collection and coding enabled thick descriptions [38]. We used Quirkos data management software (version 2.5.2) to support our data analysis. Our initial coding produced a striking contrast in participant actions: a reluctance to provide examples where they may have contributed to adverse outcomes or poor behaviour on the unit, with a facility in providing examples of their colleagues having done so. Using this finding, we began our focused coding which advanced our theoretical direction and allowed us to focus on what our team began to describe in our meetings as “dissonant schemas” across professions.

This marked the beginning of a shift into a more conceptual analysis, wherein LC used diagramming to illustrate the relationship between our categories, which were developed in further team meetings. The overwhelming interpretation of these diagrams was one of reflected, or mirrored, schemas built from our initial gerunds, which centralized the idea of dissonance because the self-schemas held conceptualizations that were in opposition to the person-schemas described by their interprofessional colleagues. The mirror diagrams spurred our team to “[re]conceptualize material that we had under-theorized” [40, p. 145]. We identified these dissonant schemas as likely having theoretical underpinnings from social psychology and turned to this literature to explore theories involving failure, success, blame, and ego. This was our CGT “flash of insight” moment, as described by Charmaz [40], wherein our coding illuminated more than the data from which it was constructed. After identifying the self-serving bias theory in the literature as one that resonated strongly both with our initial coding as well as the experiences of the research team members who provide intrapartum care in interprofessional team settings (HC, LC, TT, and AA), we added a deductive approach to our data analysis using the self-serving bias theory as a sensitizing concept [40]. Thus, a round of deductive coding was conducted by LC to determine if our theorized concepts were in line with the underlying cognitive and motivational processes consistent with self-serving biases. Our interview guides were further refined, with the goal of creating robust, theoretically adequate categories to describe the dissonant schemas using theoretical sampling strategies [40]. This theoretical sampling allowed us to take our initial conceptualizations to participants directly and ultimately informed the final version of our visual model.

Our data collection and analysis continued until theoretical sufficiency was reached [41]. We strived for the clarity, precision, and coherence that both Dey and Charmaz defined as necessary for describing our theory: our research question focused on a non-homogenous population across multiple professions, which necessitated interviewing a larger number of participants; the model we were constructing was conceptually complex and so we conducted additional interviews to ensure that our categories felt robust; we concluded interviews when our team no longer felt that they were adding new properties to our model nor leading us in other directions [40]. An audit trail was commenced in October 2021 when initial discussions regarding the project began between LC, RP, and TT. Reflective journaling and memo-writing comprised important aspects of ongoing reflexivity practice.

The study was approved by the Western University Human Studies Research Ethics Board (116210). Ethical considerations with respect to the role that LC, HB, and TT have as insiders, staff, and leaders within the birthing unit were carefully considered, and processes were in place that ensured that the identity of learners in the study was protected from anyone potentially involved in their evaluation.

## Results

**Figure 1:**
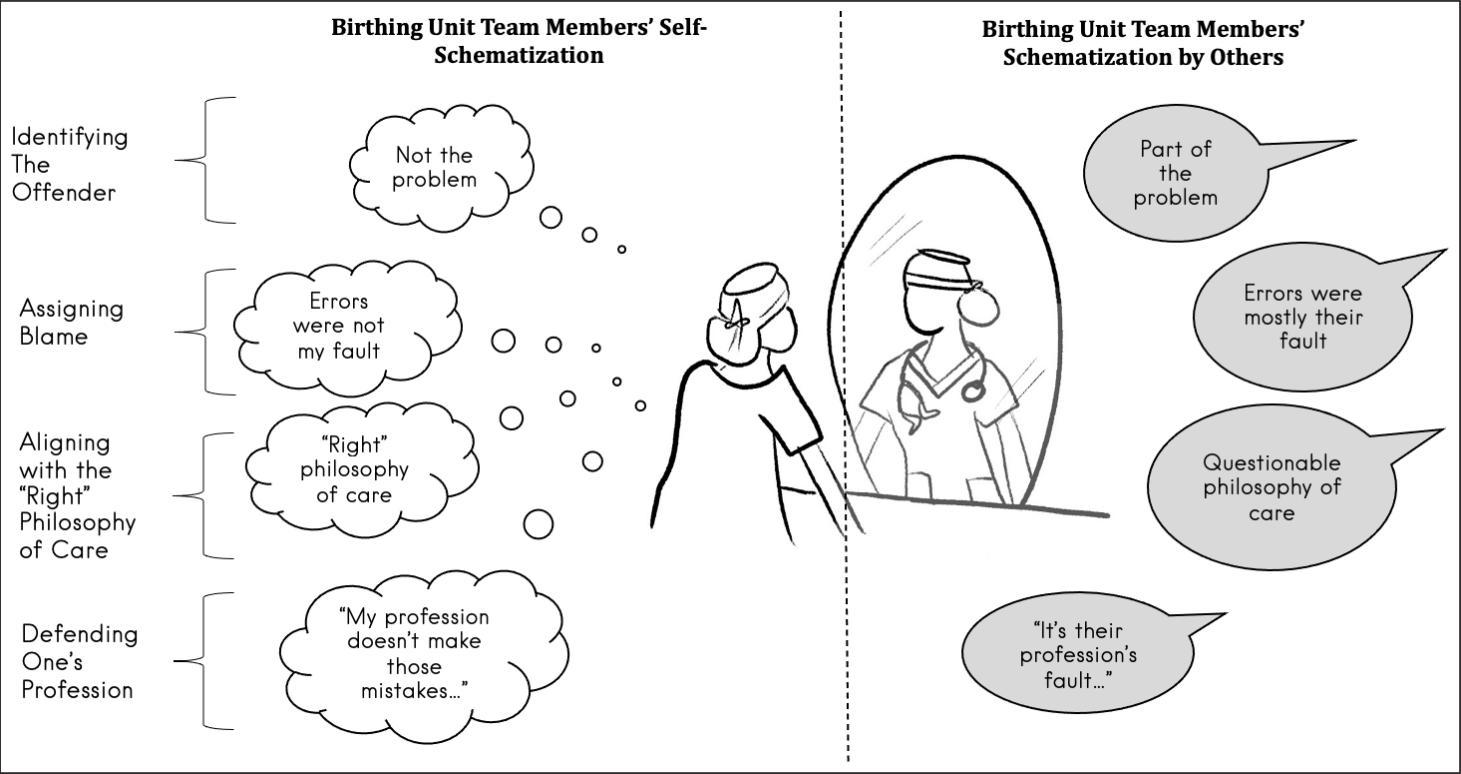
Visual model of Self- vs. Person-Schemas that birthing unit team members may create, which demonstrates the difference across four categories of information in how they may self-schematize in contrast to their colleagues’ person-schemas about them.

Birthing unit staff across all five professions constructed a self-schema of their role in FHS management that was more flattering than the person-schema created by their colleagues about them. The schemas encoded four categories of information that included (1) identifying the offender, (2) assigning blame, (3) aligning with the “right” philosophy of care, and (4) defending one’s profession (Figure 1). These categories of information represent distorted perceptual processes described by self-serving bias theories. Each of the categories will be elaborated upon with representative, verbatim quotations from the participant interviews, labelled with de-identified participant code (RM## = midwife; FM-OB## = family medicine-obstetrics; OB## = obstetricians; OB-R## = obstetrics & gynaecology residents; RN## = registered nurses).

### Identifying the offender: Not the problem/Part of the Problem

In this category of the schema framework, individuals tended not to view themselves as someone who contributed to the tensions around FHS interpretation and response. “I try to communicate in a more listening way, and explanatory way, instead of just like a this-is-what-I-want way […] like, I’m the doctor, do it. I would never approach it that way” (OB-R04). These comments were contrasted by the opposing schematic constructions of colleagues:

When the senior resident came in, [they] said, ‘I’m going to do some fetal scalp stim[ulation] to make you happy, or else you guys aren’t going to stop bugging me’. And I was like, okay […] that’s a huge warning to me like, I’m not safe to come to with your concerns. (RN04)

This schematic category was reinforced even by observed behaviours on the unit and was often broadly applied to an entire profession: “I think when I first started, I thought, you know, the OBs don’t like us, and then I saw them working with the nurses and then I thought, okay, they don’t like anybody” (RM06). In some instances, participants invoked both sides of this category within the same description:

When it comes to the midwives, I’ve taken time to genuinely formulate respectful relationships with them because as a junior resident I had a very negative experience. And then negative feedback was given about me to one of my staff and I felt like it wasn’t fair. (OB-R01)

In this case, the resident views themself as someone who is actively working to cultivate respectful interprofessional relationships (not part of the problem) but points out problematic behaviour by someone from that profession (part of the problem), emphasizing both sides of the self-serving bias.

### Assigning Blame: Errors were not my fault/Errors were mostly their fault

This category of responses depicts the asymmetric attribution of errors in FHS interpretation by individuals. When asked in the interviews if they could recall a time when they had incorrectly interpreted FHS, participants overwhelmingly responded in the negative and could not recall examples of their own errors. Responses included: “There’s nothing that actually comes to mind” (OB02), “I can think of a few bad outcomes, but was it my interpretation of the strip?” (FM-OB03), and simply “No” (RM06). A nursing participant even described the comfort they derived after the team absolved themselves of responsibility following a review of the FHS tracing in the case of a poor infant outcome:

We all look at the tracing together and we’re like, no […] we wouldn’t have acted any differently. Why this baby came out this way, we’re not sure. When you can do that, it’s really nice, because otherwise, you go home, and you just worry. (RN01)

However, when asked about errors in FHS interpretation in general, participants were quick to recall instances where other providers, generally outside their profession, had misinterpreted FHS or had been the cause of a bad outcome:

So, then the tricky thing is you come in and you get called at the last minute and you’re looking at a fetal heart rate tracing that has been a disaster for two hours and thinking why didn’t you call me about this? And it’s like, ‘well I didn’t want to bother you, it didn’t look that bad, we figured we would try X, Y, or Z first.’ (OB01)

One of the things that bothers me the most, that raises red flags for me the most is being asked to provide support for fetal heart rate interpretation, and when the request comes, the language that is used does not match what is happening in the fetal heart strip. That is one of the things that gives me the biggest red flag because now I know that whoever is monitoring the fetal heart strip may not have the skill or capacity to do so in a safe way. (OB02)

A family physician-obstetrician constructed the same schema, but from the perspective of someone consulting: “That was my first experience […] of feeling that the way that the OB consultant responded was not in keeping with the way that I thought the tracing needed to be responded to” (FM-OB01). They noted the anxiety that arose from feeling that the obstetrician with whom they had consulted did not appear to appreciate the urgency of the situation, which ultimately led to a poor outcome.

Given that both clinical reports and scholarly publications have identified issues with FHS interpretation and response as a significant contributor to preventable poor perinatal outcomes [1,10,42], the responses from study participants with respect to identifying their involvement in FHS errors was at odds with the existing literature on FHS and adverse perinatal outcomes.

### Aligning with the “Right” Philosophy of Care: “Right” philosophy of care/Questionable philosophy of care

Participants also constructed a flattering self-schema when it came to describing their own profession’s philosophy or approach to care provision that was typically at odds with the person-schema constructed by colleagues. Obstetricians, in particular, questioned the midwifery philosophy of care: “I find sometimes with the midwifery model of care, the birth person’s comfort and autonomy and wishes often would supersede what is the lower risk choice for the fetus, so that can be challenging” (OB01), while several nursing participants assigned negative views of the physician philosophy of care:

I feel like some people think it’s about money, which is really sad, but every patient is a bill. Maybe it is the more medicalized birth too. That’s what they’re taught in school. It’s a medical birth. We can make people happy because of medicine, as opposed to physiologic birth (RN-04).

While midwives staunchly defended their philosophy of care, they also questioned the motives of their obstetrical colleagues: “It’s just such a fascinating idea that you can force someone to do something rather than, how do we work within the realm of this person’s decision?” (RM01). This fundamental disagreement around bodily autonomy was then reflected in a resident comment: “And in the midwifery client model, it’s a bit like the customer is always right, they get a lot of autonomy, their clients, in what they want for care, even if it’s not necessarily the best for them” (OB-R01).

A Family Physician described the impact of what they felt were dissonant stories projected by nursing onto their profession:

[The nurse] will comment, ‘Well I’m the one who is here to advocate for the patient and look out for her best interests’. And I’m like, ‘Well what am I here for, do you think that our motives don’t align because that’s also why I’m here’. So, when I hear a comment like that, I’m like, hold on, I left my family behind on a night that I’m not on call to come look after my patient through the night, so I take that pretty personally (FM-OB04).

### Defending One’s Profession: My profession doesn’t make those mistakes/It’s their profession’s fault

Lastly, participants’ views strongly upheld professional ingroup/outgroup thinking, where they tended to see the attributes of their own profession as positive, and those of other professions as negative when it came to assigning blame for perinatal outcomes: “I think a lot of times the nurses aren’t as invested in a vaginal delivery as we sometimes are. That’s sometimes the way I feel when a tracing is being over-called and there are just some earlies [early decelerations] and I’m not concerned about them at all” (FM-OB04). In contrast to this self-schema, a nursing participant questioned the physicians’ adherence to their own guideline:

From a nursing perspective, when it comes down to litigation, that’s the guideline that we have to follow. And that is really becoming a problem, because one group of colleagues are trying to follow the protocol and the guideline by the SOGC [Society of Obstetricians and Gynaecologists of Canada], and the people that are actual obstetricians don’t want to follow that guideline. (RN05)

The tendency to easily assign errors to other professions was even noted by members outside these professions, such as the nurse who observed the schemas created between OB and midwifery: “I was a little taken aback by the toxic relationship. I know that sounds really extreme, but there’s just always comments being made at the desk about the midwife’s strip” (RN04).

In this intervention-primed study of birthing unit team members, we identified that participants create self-schemas that are ultimately more flattering than the person-schemas created by their colleagues, and that these schemas are encoded with four distorted perceptual processes. The schemas reveal dissonant stories about an individual and a profession’s role in creating a competent team and in preventing adverse perinatal outcomes.

## Discussion

When faced with challenging intrapartum FHS scenarios, birthing unit team members appeared to follow a pattern of schematization wherein they created two dissonant schemas about themselves and about others. FHS case scenarios were a relevant priming intervention for the design of this study because self-serving biases have been shown to increase concurrently with the importance of the outcome, with physiological arousal, and even despite strong expectations to be honest [29]. These conditions are an inherent reality of FHS decision-making and may explain why birthing unit teams and other health care teams operating under similar high-stakes conditions are particularly vulnerable to asymmetric schematizations between individuals and professions.

### Implications for FHS Training Programs and Quality Improvement

Self-serving bias theory provides a useful lens to understand why individuals are willing to accept responsibility for their successes, but deflect liability for adverse outcomes for which they are equally liable [32]. This strategic information processing then produces memory biases that operate in the service of self-enhancement [32]. Our results provide a framework to explore how this psychological phenomenon may act as a barrier to the accurate self-assessment of one’s inevitable contributions to preventable poor perinatal outcomes and could have significant implications in the context of quality improvement initiatives in healthcare. Even after providing an interprofessional refresher course for all staff, the need to employ self-serving biases in this team setting prevented most of the participants in our study from acknowledging that they, and members of their own professions, do commit errors. Since humans are typically not aware when they are self-enhancing, this could explain why study participants did not reflect upon the potential existence of dueling schemas and held their own schematizations as truth.

While this bias may have a functional role in protecting the birthing unit team members’ self-esteem and ego, it may also hinder performance over time and impair interprofessional social relations [31]. Quality improvement initiatives suffer when social relations are impaired because this creates weaker team identities and can lead to information-withholding towards outgroup members. This tension then “constrains the breadth of knowledge available to the teams as well as the extent to which disparate knowledge sources will be integrated through constructive interprofessional interaction” [16, p. 1328]. This is an inherent challenge to holding FHS training programs in IPE settings and needs to be considered at the outset of the training program’s creation. FHS training courses should be embedded with a compassionate understanding that being asked to confront your own role in adverse outcomes is a fundamental hardship of being a health care provider—one that impacts self-esteem and thus feelings of self-worth. Components that may protect against this, such as highlighting authentic cases of clinical excellence seen across the spectrum of birthing unit team professionals [43] could minimize the need to self-enhance and increase the feeling of being a worthy “patient’s team” member [44]. This requires a paradigmatic shifting of attitudes about error and adverse outcomes towards an organizational culture that views addressing adverse outcomes as a strength rather than an act steeped in shame and fear of reprisal. Significant investments in psychological safety as well as a high-level commitment to a quality culture would be essential facilitators to this attitudinal shift.

### Implications for Team Competence

Our analysis suggests that participants made ultimate attribution errors and regarded negative acts by their out-groups and positive acts by their in-group as dispositional and not a product of external factors [33]. This is problematic for birthing unit teams which can be composed of an evolving rotation of five or more different professions at any given time, and whose competence is determined by their social interactions with each other [45]. In creating person-schemas that routinely deflected blame for poor outcomes onto other professions, individual birthing unit team members are primed to take an adversarial stance against team members from other professions. This behaviour causes tension on the team and could hinder attempts to engage in collaborative “knotworking” [12,46]. The success of knotworking relies on trust and team members “understanding each other quickly, coordinating efforts seamlessly, and sharing control of situations fluidly” [12, p. 35] which we suggest could be impeded by the person-schemas created by participants in our study.

Strong ingroup cohesion was cited positively by many of our interview subjects about their own professions. Previous studies have highlighted the perceived ingroup benefits to ‘therapeutic bashing’ or ‘othering’ of outgroups, and the concept of intergroup conflict creating ingroup cohesion [18,19,45]. Our results add to this work by demonstrating that this method of building ingroup self-esteem through positive comparison against outgroups may be based on distorted perceptual processes that arise from self-serving biases. This affirmed the one-dimensionality that occurs in teams when members actively minimize and even deride the contributions and core values of their interprofessional colleagues [45]. Despite these behaviours, our data analysis revealed professionals who were deeply committed to their patients and their outcomes, even if their philosophies regarding this care could get in the way of achieving that object in a team setting. Training programs should be transparent about these philosophical differences (and their educational origins), while highlighting the synergy that can occur when individuals with different skillsets form a competent team, which could include feedback from patients who received excellent care from an interprofessional team. This would increase the psychosocial fidelity of the FHS case scenarios.

### Implications for FHS Training Programs as IPE: Forming a Team Identity

Previous research has emphasized that successful interprofessional collaboration requires expanding one’s identity beyond the borders of one’s profession to a broader “patient’s team” definition [11,14,16,19]. One method through which this can be achieved involves superordinate goal setting as a mechanism through which disparate groups can overcome their self-serving attribution errors [15,16,47]. Unfortunately, in this birthing unit team setting, our results showed that differences in philosophies of care deeply impact what may, at first glance, have appeared to be an obvious superordinate goal (the best possible perinatal outcome for both the birthing person and the neonate). Participants in our study may struggle to shape superordinate goals with the other professions, because the deeply divisive philosophies of care and differing cognitive maps hindered their ability to do so. Our interviews revealed that the health of the fetus was sometimes weighed against the well-being of the parturient, and upholding patient autonomy was viewed as equivalent in importance as the health outcomes of the dyad by participants from one profession, but not another. Unit efficiency, financial gain, patient satisfaction, adherence to protocols, and time to delivery complicated the development of superordinate goal setting, which may prove too simplistic for the complexities of a busy birthing unit. This finding adds to previous research that explored the challenges teams face in achieving a shared object [12,19]. The social, cultural, and historical considerations of unit and team rules, communities, and divisions of labour can complicate a team’s ability to achieve a superordinate goal [12], and the findings in this study demonstrate that these same factors also prevent birthing unit teams from even forming the superordinate goal in the first place. We suggest a possible different direction for superordinate goals—one that acknowledges our inherent human psychological need to protect our self-esteem and to foster feelings of self-worth from our roles on a health care team. This could define a new team identity, one that is based on a common belief that members are deserving of this protection and want this protection for themselves, emphasizing the interdependent nature of the team members and this goal.

Our suggested framework builds on the current understanding of the psychosocial behaviours of individuals in interprofessional health care teams and highlights the significant barriers that these dissonant schemas represent for team competence and for the delivery of effective FHS training programs. To address the necessary improvement to these programs, this framework could be presented both explicitly in the didactic and discussion-based portions of the training, as well as incorporated into FHS case scenarios. Training sessions could facilitate the recognition that humans are prone to employing self-serving biases in high-stakes situations such as FHS interpretation and response, but that they are rooted in distorted perceptual processes. Participants may then begin to engage in important discussions on how to modify the self- and person-schemas they have created. Though modifying schemas can be uncomfortable and involves a commitment to changing errors in thinking, FHS training programs could provide an environment supported by facilitators and peers who are collaboratively engaged in this process. Programs could also include mechanisms to reduce the other primary reason that individuals resort to self-serving biases, which is their motive to enhance self-esteem [44].

## Methodological Strengths and Limitations

Strengths of this study are represented in the rich diversity of sampling across all five professions that form the basis of many birthing unit teams around the world. The main limitation—that study participants were recruited from a single site—does not necessarily limit the transferability of the findings as the international literature addressing interprofessional team dynamics describes similar challenges in FHS decision-making in a variety of birthing unit settings. Transferring these findings to other birthing units depends largely on the local culture and context of the unit. The limitations of our decision to interview subjects at a single site were weighed against the potential methodological benefits of the serendipitous timing to run the priming intervention (FHS refresher courses) for all members of the birthing unit, which we felt represented a unique study design opportunity and contributed to the richness and depth of this intervention-primed CGT study [36].

## Future Research Directions

Identifying the psychological reasoning behind the use of self-serving biases on birthing unit teams serves as a useful starting point to begin to address mitigation strategies. Future research in team settings that examines ways to protect the ego and bolster self-esteem—both necessary to function in stressful, high-stakes settings [44]—without relying on the need to self-enhance or make ultimate-attribution errors through harmful attitudes towards outgroup members, could prove useful in breaking down professional silos and creating a common team-based identity [11,14]. Further research must focus on the practical steps to tackle the challenges of self-serving biases and ensure the optimization of interprofessional teams in learning and in practice.
